# A tonsillar location of a malignant schwannoma: a case report

**DOI:** 10.1186/s13000-022-01226-3

**Published:** 2022-05-30

**Authors:** Imane Boujguenna, Hind Ousehal, Atmane Zaroual, Mohamed E. L. Bouderkaoui, Abdelaziz Raji, Chihab Bouyaali, Najat Cherif Idrissi El Ganouni, Anass Fakhri, Hanane Rais

**Affiliations:** 1 Department of Pathological Anatomy, FMPM-UCA-CHU Mohamed VI, 40000 Marrakech, Morocco; 2 Department of Otolaryngology, FMPM-UCA-CHU Mohamed VI, 40000 Marrakech, Morocco; 3 Radiology Department, FMPM-UCA-CHU Mohamed VI, 40000 Marrakech, Morocco

**Keywords:** Malignant schwannoma, Tonsil, Pathological anatomy

## Abstract

**Introduction:**

Malignant schwannoma is a malignant tumor of differentiation of Schwann cells or perineural cells.

**Observation:**

The patient was a 74-year-old woman with no particular pathological history. She presented swallowing difficulty of solids and odynophagia, evolving for 1 year. Physical examination revealed a budding tumor of the left palatine tonsil without cervical adenopathy. The CT scan confirmed the lesions and the absence of tumor extensions. Histological and immunohistochemical examination of the biopsy sample of the tonsil tumor concluded to be a malignant schwannoma. The patient underwent a tonsillectomy with postoperative follow-up.

**Discussion:**

Malignant schwannomas are aggressive tumors. They usually occur in young adults. They mainly affect nerves and soft tissues. Occurrence in the amygdala is rare.

**Conclusion:**

The association of malignant schwannoma of the palatine tonsil and advanced age is rare.

## Introduction

Malignant schwannoma is a malignant tumor with evidence of Schwann cell or perineural cell differentiation, arising in nerves and soft tissues (WHO 2017).

We report a rare localization of a schwannoma in the tonsil in a 74 year old woman without pathological history.

### Observation

The patient is a 74-year-old woman with no pathological history, presented with a discomfort in swallowing solids with odynophagia and weight loss evolving for 1 year. The physical examination found a patient with stable vital signs and showed a budding tumor of the left palatine tonsil without cervical adenopathy. The CT scan confirmed the lesion and the absence of tumor extension.

**Fig. 1:**
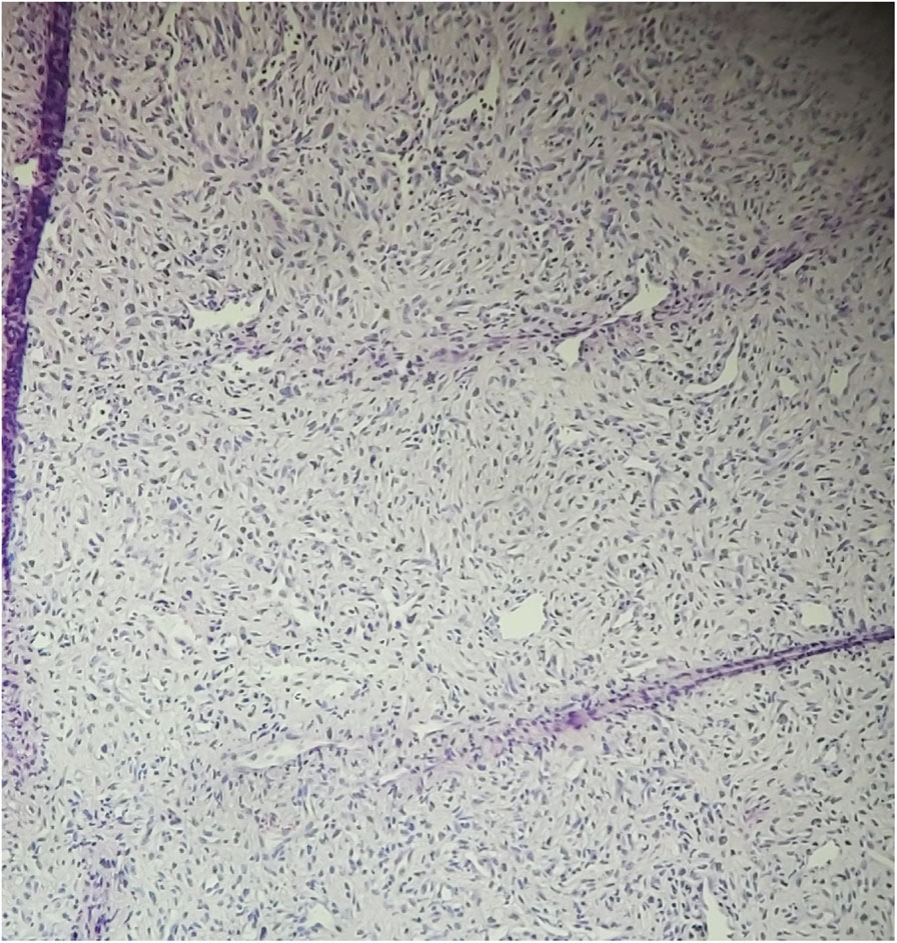
Mesenchymal proliferation with a neurogenic appearance HEx20

**Fig. 2:**
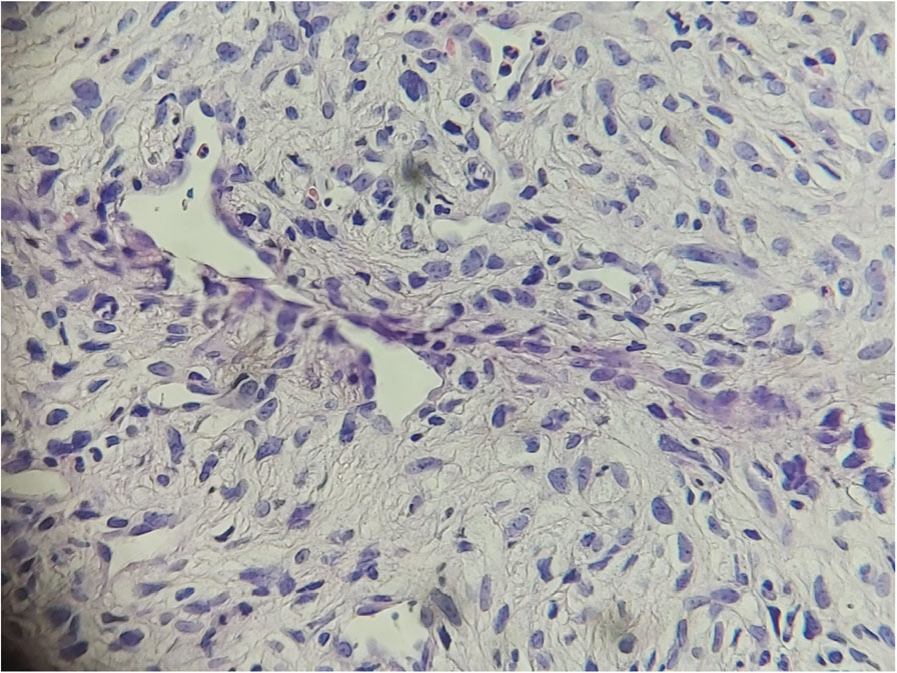
storiform organization of HEx40 tumor proliferation

**Fig. 3:**
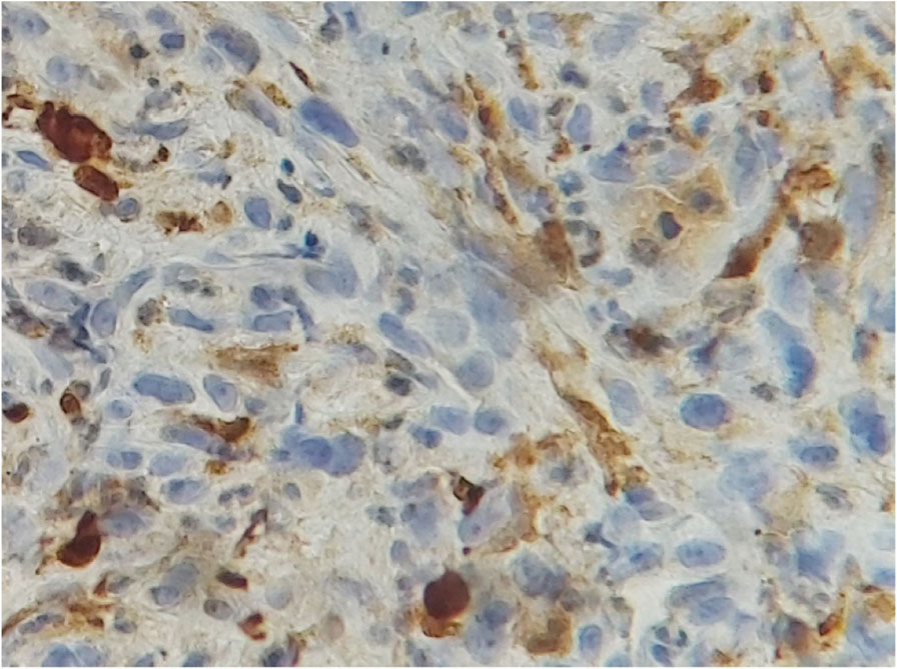
Moderate partial positivity of tumor cells of PS100 (patchy)

**Fig. 4:**
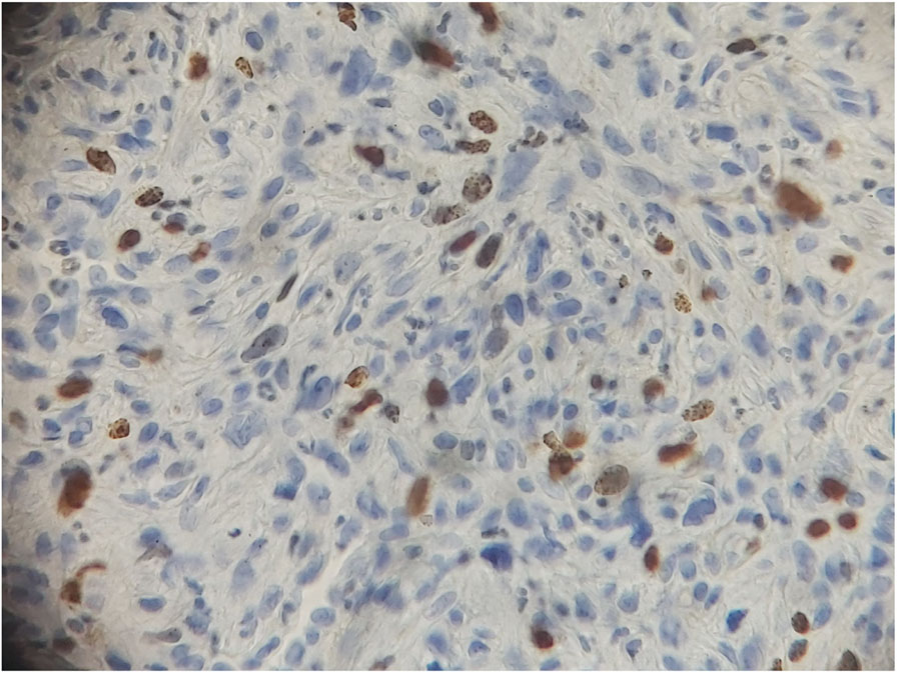
Nuclear expression of 30% of tumor cells of the anti Ki67 antibody

A biopsy was performed, and the histological examination found a tumor proliferation arranged in intersecting bundles, sometimes hypocellular, sometimes hypercellular with coils and storiform arrangements. It is made of large, plump, spindle-shaped cells with variable atypia, the number of mitoses is 6 per 10 fields at high magnification, counted in different areas (Figs. 1, 2). There was infiltration border. The immunohistochemical study shows a moderate partial positivity of tumor cells of PS100 (patchy) and an absence of expression of tumor cells of CK, P63, CD45, HMB45 and Melan A. Ki67 was expressed by 30% of tumor cells (Figs. 3, 4). This led to the diagnosis of malignant schwannoma.

The patient underwent a tonsillectomy with postoperative follow-up.

## Discussion

Primary peripheral nerve tumors account for 1 to 2% of soft tissue tumors. They can be benign or malignant, usually seen in the setting of Von Recklinghausen disease [1, 2].

They occur in young adults and mainly affect nerves and soft tissue. Occurrence in the tonsil is rare [3, 4]. About 25–48% of schwannomas occur in the head and neck region, but only 1% have an intra-oral origin. Schwannoma of the tonsils is extremely rare and, to our knowledge, only 11 other reports of tonsillar schwannoma have been published: (Table 1).

**Table 1:** Table 1 Published cases of tonsillar schwannoma

Reference and year	Sex/Age (years)	Presentation	Localisation	Histology	Treatment	Follow-up
Present report	Female/74	DWS	LT	Malignant	TTT	NED
Pran Gopal Datta and al, 2020 [5]	Male/42	DWS	LT	Benign	TT	NED
LING-XIANG RUAN and al, 2008 [6]	Male/37	IFM, snoring	RT	Benign	TTT	NED at 6 months
Anil and al, 2005 [7]	Male/38	DWS, OO, RP to the left ear	LT	Benign	TT	NED at 18 months
Dai and al, 2003 [8]	Male/34	Pharyngodynia, OO, RP, bloody phlegm	RT	Benign	TTT	NED at 1 year
zhou and al, 2003 [9]	Female/28	DWS, OO	LT	Benign	TTT	–
Bildirici and al, 2002 [4]	Female/69	IFM, no other complaints	RT	Benign	TT	NED at 1 year
Guo et al., 2000 [10]	Male/20	Foreign body feeling, haemoptysis, OO	LT	Benign	TTT	–
Lall and al, 1999 [11]	Female/13	DWS		Benign	TT	NED
Xu and Li, 1998 [12]	Female/62	Foreign body feeling	LT	Malignant	TTT	NED at 4 months
Wu et al., 1992 [13]	Male/34	IFM, resected two times, recurred two times	LT	Malignant	TTT	Died after 4 months
Naik and Agrawal 1975 [14]	Male/45	Foreign body feeling	RT	Benign	TT	–

*IFM* Initially found mass, *DWS* Difficulty with swallowing, *OO* Odynophagia, *RP* Radiating pain, *RT* Right tonsil, *LT* Left tonsil, *TT* Tumourectomy, *TTT* Tonsillectomy plus tumourectomy, *NED* No evidence of disease

Malignant schwannomas are mesenchymal tumors that develop at the expense of peripheral elements of nerve endings: SCHWANN sheath and intramural or subserosal plexuses [1, 2].

These tumors do not have pathognomonic clinical or radiological features. This makes the confirmatory diagnosis based essentially on standard and complementary anatomopathological examinations [3].

Histologically, it is a proliferation of large, plump, spindle-shaped cells with variable atypia, the number of mitoses is highly variable (usually > 4/10 fields) counted in different areas. The arrangement is variable, most often in intersecting bundles, with swirls, storiform aspects, or even rarely outline nuclear palisades. Hypercellular bundles may be present next to hypocellular, myxoid bundles, having lost the parallel arrangement of the nuclei. More rarely, storiform or loosely coiled arrangement mimicking tactoid bodies, sometimes perivascular densification [1, 2, 4, 11].

Neoplastic cells express PS100 in 50–70% of the cases, negativity for HMB45 and melan-A allows to rule out a melanoma [1, 4].

The histological diagnosis of malignant schwannomas of rare location presents two main problems:

-Differential histological diagnosis between schwannoma, on one hand, from other mesenchymal tumors is difficult, based on immuno-histochemical criteria: cells of nervous origin express PS100. On the other hand, distinction from melanoma is assessed by the absence of expression of Melan A and HMB45. This was also the case in our observation.

-Affirmation of the benign or malignant nature of the tumor: because mitoses, nuclear abnormalities and cellular polymorphism with high expression of Ki67 in tumor cells are not always present [1, 4, 15].

Sometimes only the evolution and the appearance of metastases allow to affirm malignancy.

Treatment consists of complete surgical removal of the tumor [2, 4, 7, 11].

The prognosis of these tumors is variable, and the recurrence rate depends on the surgical resection. After surgery, the survival is 79% if resection is complete, 22% if resection is impossible or in metastasis [1–3, 7].

## Conclusion

Malignant schwannoma is an aggressive tumor with a poor prognosis. It poses a problem of positive diagnosis especially in case of atypical localizations or in the absence of underlying conditions.

## Data Availability

Data Available.
